# Comparison of Vendor-Pretrained and Custom-Trained Deep Learning Segmentation Models for Head-and-Neck, Breast, and Prostate Cancers

**DOI:** 10.3390/diagnostics14242851

**Published:** 2024-12-18

**Authors:** Xinru Chen, Yao Zhao, Hana Baroudi, Mohammad D. El Basha, Aji Daniel, Skylar S. Gay, Cenji Yu, He Wang, Jack Phan, Seungtaek L. Choi, Chelain R. Goodman, Xiaodong Zhang, Joshua S. Niedzielski, Sanjay S. Shete, Laurence E. Court, Zhongxing Liao, Fredrik Löfman, Peter A. Balter, Jinzhong Yang

**Affiliations:** 1 Department of Radiation Physics, Division of Radiation Oncology, The University of Texas MD Anderson Cancer Center, Houston, TX 77030, USA; xchen20@mdanderson.org (X.C.); yzhao15@mdanderson.org (Y.Z.); hbaroudi@mdanderson.org (H.B.); mdel2@mdanderson.org (M.D.E.B.); adaniel1@mdanderson.org (A.D.); sgay1@mdanderson.org (S.S.G.); yu.cenji@mayo.edu (C.Y.); hewang@mdanderson.org (H.W.); xizhang@mdanderson.org (X.Z.); jsniedzielski@mdanderson.org (J.S.N.); lecourt@mdanderson.org (L.E.C.); pbalter@mdanderson.org (P.A.B.); 2 The University of Texas MD Anderson Cancer Center UTHealth Houston Graduate School of Biomedical Sciences, Houston, TX 77030, USA; sshete@mdanderson.org; 3 Department of Radiation Oncology, Division of Radiation Oncology, The University of Texas MD Anderson Cancer Center, Houston, TX 77030, USA; jphan@mdanderson.org; 4 Department of GU Radiation Oncology, Division of Radiation Oncology, The University of Texas MD Anderson Cancer Center, Houston, TX 77030, USA; stchoi@mdanderson.org; 5 Department of Breast Radiation Oncology, Division of Radiation Oncology, The University of Texas MD Anderson Cancer Center, Houston, TX 77030, USA; crgoodman@mdanderson.org; 6 Department of Biostatistics, Division of Discovery Science, The University of Texas MD Anderson Cancer Center, Houston, TX 77030, USA; 7 Department of Thoracic Radiation Oncology, Division of Radiation Oncology, The University of Texas MD Anderson Cancer Center, Houston, TX 77030, USA; zliao@mdanderson.org; 8 RaySearch Laboratories AB, 113 68 Stockholm, Sweden; fredrik.lofman@raysearchlabs.com

**Keywords:** auto-segmentation, commercial segmentation, radiotherapy, U-Net

## Abstract

Background/Objectives: We assessed the influence of local patients and clinical characteristics on the performance of commercial deep learning (DL) segmentation models for head-and-neck (HN), breast, and prostate cancers. Methods: Clinical computed tomography (CT) scans and clinically approved contours of 210 patients (53 HN, 49 left breast, 55 right breast, and 53 prostate cancer) were used to train and validate segmentation models integrated within a vendor-supplied DL training toolkit and to assess the performance of both vendor-pretrained and custom-trained models. Four custom models (HN, left breast, right breast, and prostate) were trained and validated with 30 (training)/5 (validation) HN, 34/5 left breast, 39/5 right breast, and 30/5 prostate patients to auto-segment a total of 24 organs at risk (OARs). Subsequently, both vendor-pretrained and custom-trained models were tested on the remaining patients from each group. Auto-segmented contours were evaluated by comparing them with clinically approved contours via the Dice similarity coefficient (DSC) and mean surface distance (MSD). The performance of the left and right breast models was assessed jointly according to ipsilateral/contralateral locations. Results: The average DSCs for all structures in vendor-pretrained and custom-trained models were as follows: 0.81 ± 0.12 and 0.86 ± 0.11 in HN; 0.67 ± 0.16 and 0.80 ± 0.11 in the breast; and 0.87 ± 0.09 and 0.92 ± 0.06 in the prostate. The corresponding average MSDs were 0.81 ± 0.76 mm and 0.76 ± 0.56 mm (HN), 4.85 ± 2.44 mm and 2.42 ± 1.49 mm (breast), and 2.17 ± 1.39 mm and 1.21 ± 1.00 mm (prostate). Notably, custom-trained models showed significant improvements over vendor-pretrained models for 14 of 24 OARs, reflecting the influence of data/contouring variations in segmentation performance. Conclusions: These findings underscore the substantial impact of institutional preferences and clinical practices on the implementation of vendor-pretrained models. We also found that a relatively small amount of institutional data was sufficient to train customized segmentation models with sufficient accuracy.

## 1. Introduction

In radiation therapy, precise delineation of both clinical target volumes (CTVs) and organs-at-risk (OARs) is essential for achieving effective treatment outcomes. A routine task in the current standard of care, manual segmentation of CTVs and OARs, is not only time- and labor-intensive but also prone to inter- and intra-observer variability [1,2,3,4,5,6]. Moreover, the time-intensive nature of manual segmentation poses a significant bottleneck in the context of adaptive radiotherapy, which requires delineating CTVs and OARs daily to improve target coverage and normal-tissue sparing [7]. Given these challenges, the use of automated segmentation techniques has attracted significant attention for their potential to streamline the clinical workflow and enhance the consistency of contours [8]. The application of deep learning (DL)-based auto-segmentation algorithms in particular has shown substantial improvement over conventional methods such as atlases and region-growing techniques [9]. DL-based algorithms have shown improved accuracy, accelerated processing speed, enhanced efficiency, and increased consistency in auto-segmentation, thereby propelling their integration into routine clinical practice in radiation therapy [8].

Although in-house DL-based algorithms can be advanced and effective for the automated delineation of CTVs and OARs [10,11,12,13], the need for technical expertise to facilitate training, integration into the clinical workflow, and maintenance of those algorithms presents a notable challenge, especially for smaller institutions that may have shortages of clinical and technical staff but would benefit significantly from the use of commercialized automated algorithms. In this context, several commercially available DL-based auto-segmentation tools have emerged that offer models pretrained by the vendor that are hosted locally or in the cloud and feature user-friendly treatment planning system (TPS) interfaces. Examples of such tools include MVision AI (MVision AI Oy, Helsinki, Finland), Limbus (Limbus AI Inc., Regina, SK, Canada), MIM Protégé AI (MIM Software Inc., Cleveland, OH, USA), and RayMachine (RaySearch Laboratories AB, Stockholm, Sweden). Clinical assessment of these commercial DL-based algorithms has shown satisfaction with regard to usability and promising enhancements in efficiency [14,15,16,17,18,19,20]. However, the performance of DL-based algorithms remains contingent upon the characteristics of the training datasets and model architectures, with notable discrepancies observed among different commercial DL-based auto-segmentation tools [21,22,23]. Moreover, cross-institutional investigations have revealed that variations in scanning devices and image acquisition protocols significantly affect the performance of DL-based algorithms [24,25]. Zero-shot object detection presents substantial challenges for model inference [26], particularly when the users and developers operate in geographically separated environments. Consequently, the applicability of commercial tools with local institutional datasets may be compromised, necessitating manual corrections that could offset the anticipated time-saving benefits [27]. Therefore, meticulous and case-specific quality assurance (QA) is imperative for successfully implementing commercial DL-based auto-segmentation tools at individual institutions [28,29]. In addition to pretrained models, certain vendors recently introduced DL-based toolkits designed to facilitate the training of custom models; once trained, these custom models can be used similarly to the pretrained models. These customized models have the potential to capture institutional preferences with regard to clinical contours [30], such as patient populations, contouring preference, and variations in image protocols, while retaining the advantages of accessibility and efficiency associated with commercial systems. The primary objective of this study was to assess the effects of data variations on the performance of DL-based segmentation models by comparing custom-trained DL-based segmentation models using vendor-supplied training toolkits with vendor-pretrained models on institutional datasets pertaining to head-and-neck (HN), breast, and prostate cancer.

## 2. Materials and Methods

### 2.1. Dataset

The use of patient data in this work was approved by the institutional review board (IRB) with a waiver of informed consent for retrospective study. All experiments were performed in accordance with relevant guidelines and regulations. Simulation computed tomography (CT) scans, along with clinically approved contours, were used to train custom models by using DL-based toolkits provided by RayMachine (RaySearch Laboratories AB, Stockholm, Sweden) and to assess both the vendor-pretrained and custom-trained models. To assess the model’s performance across diverse anatomic disease sites, CT scans, and clinically approved contours were gathered from 210 patients, 53 with HN cancer, 49 with cancer in the left breast, 55 with cancer in the right breast, and 53 with prostate cancer. The datasets were divided into training, validation, and testing sets with the following splits: 30 training/5 validation/18 testing for the HN, 34/5/10 for the left breast, 39/5/11 for the right breast, and 30/5/18 for the prostate. The clinically approved contours obtained for this study met institutional guidelines informed by both the expertise of our clinicians and established international guidelines. In contrast, the vendor-pretrained models were trained by using CT scans and contours gathered from a combination of publicly available datasets and vendor collaborating clinics according to published consensus guidelines, with slight modifications, and specifications for certain structures. Details on the number of samples used for training/validation in the vendor-pretrained and custom-trained models, plus the corresponding guidelines, are presented in Table 1. Notably, our institutional practice includes some adjustments to the referenced international guidelines, and the clinically approved contours were delineated in accordance with institutional preferences and practices. To enhance the validity of the comparison, 24 structures were selected for training custom-trained models to align with the contour availabilities in both our clinical practice and vendor-pretrained models. These structures, grouped by anatomic sites, included (1) the brainstem, spinal cord, esophagus, left and right eyes (Eye_L, Eye_R), mandible (Bone_Mandible), left and right optic nerves (OpticNrv_L, OpticNrv_R), left and right parotid glands (Parotid_L, Parotid_R), and the left and right submandibular glands (Glnd_Submand_L, Glnd_Submand_R); (2) clinical target volume of breast (CTV_Breast), ipsilateral level 1 to level 3 axillary lymph nodes (LN_Ax_L1–LN _Ax_L3), ipsilateral internal mammary lymph nodes (LN_IMN), and the contralateral breast as an OAR (OAR_Breast); and (3) prostate, rectum, bladder, and the left and right femoral heads (Femur_Head_L, Femur_Head_R).

### 2.2. DL Model Training

We implemented a two-stage 3D U-Net architecture within the DL-based toolkits from RayMachine (RaySearch Laboratories AB, Stockholm, Sweden). The two-stage localization–segmentation strategy and the 3D U-Net architecture are widely adopted in commercial auto-segmentation tools. Each model comprised a preliminary coarse localization stage that identified the positions and receptive fields of all structures within the target site, followed by a subsequent refined segmentation stage focusing on local individual structure segmentation (Table 1). For each disease site, we trained a 3D U-Net localization submodel by using a coarse isotropic resolution of 3 mm followed by independent 3D U-Net submodels trained for each individual structure, with a finer resolution ranging from 1 mm to 1.5 mm depending on structure size. The training and inference process was divided into multiple steps: (1) maximum bounding boxes were established based on body contours (localization stage) or individual structures (segmentation stage) from all training and validation patients, with an isotropic padding of 2 cm; (2) training and validation CT images and ground truth contours were preprocessed to the desired size and resolution, depending on the target structures; (3) 3D U-Net models were trained for localization (multi-labels) and segmentation (single label); (4) during inference, input volumes of the coarse localization were defined using the testing patient’s body contour and desired coarse bounding box size from step 1, with automatic padding; (5) the localization model conducted a coarse segmentation to localize different structures; (6) input volumes were refined for individual structures using coarse segmentation results and fine bounding box sizes from step 1, with automatic padding; (7) the model performed refined segmentation for individual structures.

The 3D U-Net models used for both localization and segmentation employed a four-level contracting-expansive architecture. The contracting path consisted of repeated application of two 3 × 3 × 3 convolutions, each followed by an instance normalization layer and a rectified linear unit (ReLU) activation. A 2 × 2 × 2 max pooling layer was used for down-sampling, followed by a dropout layer with a 90% probability. Each step in the expansive path involved 2 × 2 × 2 transpose convolutions with a stride of 2, followed by two 3 × 3 × 3 convolutions. The layers in the contracting path were skip-connected and concatenated with layers in the expansive path within the same level. The number of features in each level was set as 24, 48, 96, and 192 (doubling in the contracting path and halving in expansive), whereas the input size varied for individual structures.

Data augmentation techniques included random translation and rotation, elastic deformation, and random cropping along the *z* axis. The learning rate was set to 0.001, with models trained for 1000 epochs. Checkpoints were saved every 10 epochs, and the best model was selected based on the training and validation DSCs to prevent overfitting. A sparse categorical cross-entropy loss function was used with a L2 regularization (weight, 1 × 10^−5^). Training was performed on an NVIDIA GV100 32G GPU from a Dell workstation (Dell inc., Round Rock, TX, USA). Depending on the target structure size and desired resolution, model training took an average of 2.7 to 5.8 h.

Similarly, the vendor-pretrained models were developed by using an identical localization–segmentation strategy and network architecture, which were released in RayStation 11B (RaySearch Laboratories AB, Stockholm, Sweden). The primary distinction between the vendor-pretrained and custom-trained models lay in the source and amount of training and validation datasets: the custom-trained model was developed by using local institutional datasets with relatively fewer training and validation samples.

### 2.3. Geometric Evaluation

Both the custom-trained models and vendor-pretrained models were applied to testing CT scans to generate auto-segmented contours. The auto-segmented contours were quantitatively assessed by comparing them with the clinically approved contours. Two distinct metrics were used to quantify the similarity between auto-segmented contours and clinically approved contours: (1) the Dice similarity coefficient (DSC); and (2) the mean surface distance (MSD), calculated as the average Euclidean distance between the surfaces of contours *X* and *Y*, measured bidirectionally. The DSC provides a dimensionless assessment of volumetric overlap, with 0 denoting no overlap and 1 indicating perfect agreement; the MSD provides a measurement of proximity in millimeters. Wilcoxon signed-rank tests were used to compare the performance of the vendor-pretrained and custom-trained models for each specific structure in terms of DSC and MSD. *p* values of less than 0.05 were considered to indicate statistically significant differences in the agreement with clinically approved contours. In breast models, only ipsilateral nodes were considered to prevent the creation of non-relevant contralateral structures. Consequently, the evaluation of the left and right breast models was performed jointly by categorizing the left and right breasts as either ipsilateral (CTV_Breast) or contralateral (OAR_Breast).

### 2.4. Dosimetric Evaluation

Clinical treatment plans that were initially created based on clinically approved contours were also used to generate dose-volume histograms (DVHs) for both the auto-segmented contours and clinically approved contours. For HN patients, the prescribed doses varied from 14 Gy in 4 fractions to 69.96 Gy in 33 fractions. Prescribed doses ranged from 26 Gy in 5 fractions to 40.05 Gy in 15 fractions for patients undergoing partial breast irradiation, and from 40 Gy in 5 fractions to 85.8 Gy in 39 fractions for patients with prostate cancer. To quantify the effects of contour differences on reported doses, we compared the mean dose and maximum dose delivered to each structure defined by the auto-segmented vs. clinically approved contours. Two metrics were calculated: (1) the error in mean dose, ΔD_mean_, defined as the difference between the mean dose represented by the auto-segmented contour and that of the clinically approved contour for a specific structure, normalized to the prescription dose; and (2) the error in maximum dose, ΔD_max_, similarly defined as the difference between the maximum dose indicated by the auto-segmented contour and that of the clinically approved contour, normalized to the prescription dose. The mean dose is particularly pertinent to parallel organs, such as parotid and submandibular glands, whereas the maximum dose is more relevant to serial organs like the brainstem and spinal cord.

## 3. Results

The calculated DSCs and MSDs for all 24 structures within three distinct anatomic sites are shown in Table 2. Corresponding boxplots are available in Supplementary Materials. Overall, the custom-trained models showed significantly improved conformity with clinically approved contours in the three different sites (*p* < 0.001). The custom-trained models performed inferiorly in two of the structures (Glnd_Submand_L and LN_Ax_L3), but differences in these two structures were not statistically significant (*p* > 0.05). In the HN dataset, the custom-trained model significantly outperformed the vendor-pretrained model for Bone_Mandible and Parotid_L in terms of both DSC and MSD; however, segmentation accuracy for other structures did not show significant differences. Within the breast dataset, all structures demonstrated significantly superior alignment with clinically approved contours in the custom-trained model except for LN_Ax_L3. In the prostate dataset, the custom-trained model exhibited superior alignment with clinically approved contours for the prostate, rectum, Femur_Head_L, and Femur_Head_R.

### 3.1. Auto-Segmentation for Head and Neck Cancer

**Figure 1:**
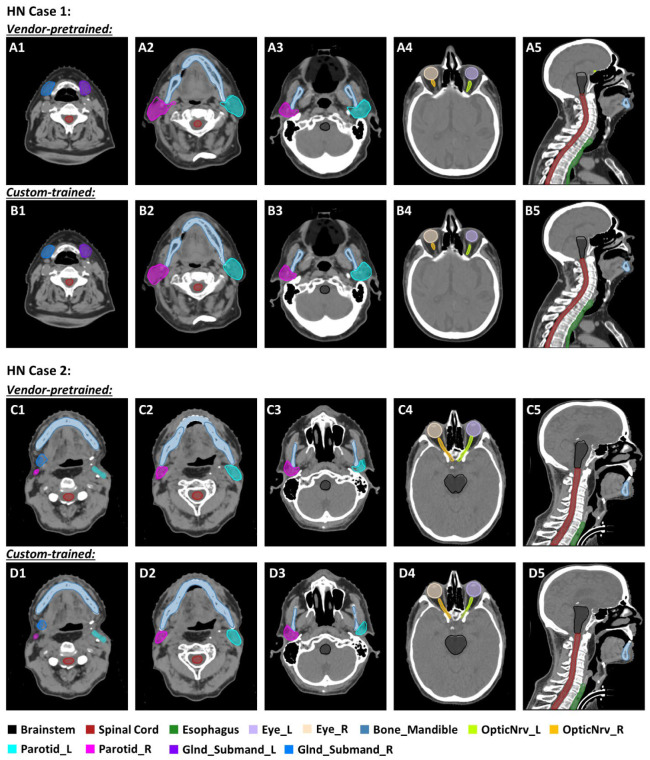
Comparison of contours derived from a vendor-pretrained head-and-neck model (Case 1, (**A1**–**A5**); Case 2, (**C1**–**C5**)) and a custom-trained head-and-neck model (Case 1, (**B1**–**B5**); Case 2, (**D1**–**D5**)). Color wash indicates clinically approved contours. Abbreviations: _L, left; _R, right; OpticNrv, optic nerve; Glnd_Submand, submandibular gland.

A comparison of the vendor-pretrained versus custom-trained models for two HN cases (Cases 1 and 2), including one “good” case and one “bad” case in terms of average DSCs from the custom-trained model, is shown in Figure 1. Clinically approved contours (the standard against which the models are being compared) are illustrated as a color wash. The contours generated by the custom-trained model for parotid and brainstem more closely resembled the clinically approved contours in both cases [Figure 1 (Case 1-B2,B3,B5; Case 2-D2–D5)]. Minor deviations are observed along the border between the mandible and teeth in the vendor-pretrained model of Case 2 [Figure 1 (Case 2-C2)]. Correctly identifying the right submandibular gland in Case 2 was difficult in both models [Figure 1 (Case 2-C1,D1)]. Notably, no discernible differences in the performance of the two models were found for the spinal cord, esophagus, eyes, and optic nerves.

### 3.2. Auto-Segmentation for Breast Cancer

**Figure 2:**
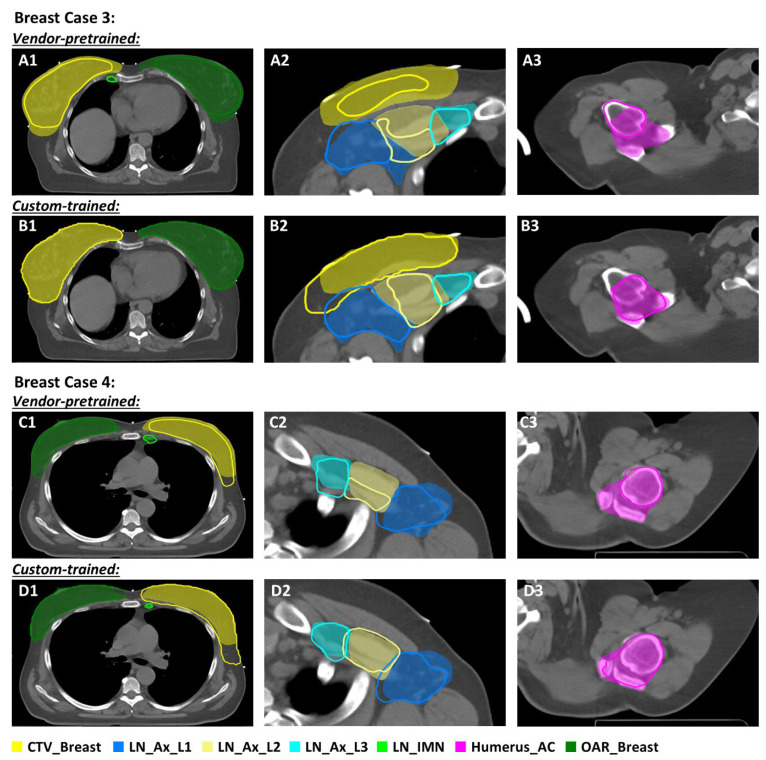
Comparison of contours derived from a vendor-pretrained breast model (Case 3, (**A1**–**A3**); Case 4, (**C1**–**C3**)) and a custom-trained breast model (Case 3, (**B1**–**B3**); Case 4, (**D1**–**D3**)). Color wash indicates clinically approved contours. Abbreviations: CTV_Breast, clinical target volume of breast; LN_Ax_L1- LN_Ax_L3, level 1 to level 3 axillary lymph nodes; LN_IMN, internal mammary lymph node; Humerus_AC, humeral head, and acromioclavicular joint.

A comparison of auto-segmented contours generated by the custom-trained model and the vendor-pretrained model for patients with breast cancer (Cases 3 and 4) is shown in Figure 2. The contours of the left and right breasts from the custom-trained model align with our former institutional practice in 3D conformal radiotherapy (3D CRT), encompassing the skin and pectoralis major muscle in the cases involving treatment of lymph nodes [Figure 2 (Case 3-B1, Case 4-D1)]. In contrast, the vendor-pretrained model adheres to guidelines that exclude the pectoralis major, and the breast contour is retracted from the skin to accommodate dose buildup within the breast in Volumetric Modulated Arc Therapy (VMAT) [Figure 2 (Case 3-A1, Case 4-C1)]. Also, the contours of LN_Ax_L2 from the custom-trained model match the clinically approved contours (color wash) more closely than the contours from the vendor-pretrained model [Figure 2 (Case 3-A2,B2; Case 4-C2,D2)]. As noted above, the differences in LN_Ax_L2 reflect a discrepancy in preferences between the vendor-pretrained and custom-trained models: the clinically approved contours incorporate axillary vessels posterior to the pectoralis minor muscle, the pectoralis minor muscle, and interpectoral (“Rotter’s”) nodes extending to the inner surface of the pectoralis major muscle, whereas the vendor-pretrained model contours encompass only the axillary vessels posterior to the pectoralis minor muscle. Thus, the vendor-pretrained model incorporated an additional contour of the interpectoral (“Rotter’s”) lymph nodes encompassing the space between the pectoralis minor and major muscles. Regarding the humeral head, our institutional preference is to create a single Humerus_AC contour to minimize the radiation dose delivered to the bone marrow in the humeral head and the acromioclavicular joint. Although the vendor-pretrained model accurately delineated the humeral head, it did not adhere to our preference to include the acromioclavicular joint.

### 3.3. Auto-Segmentation for Prostate Cancer

**Figure 3:**
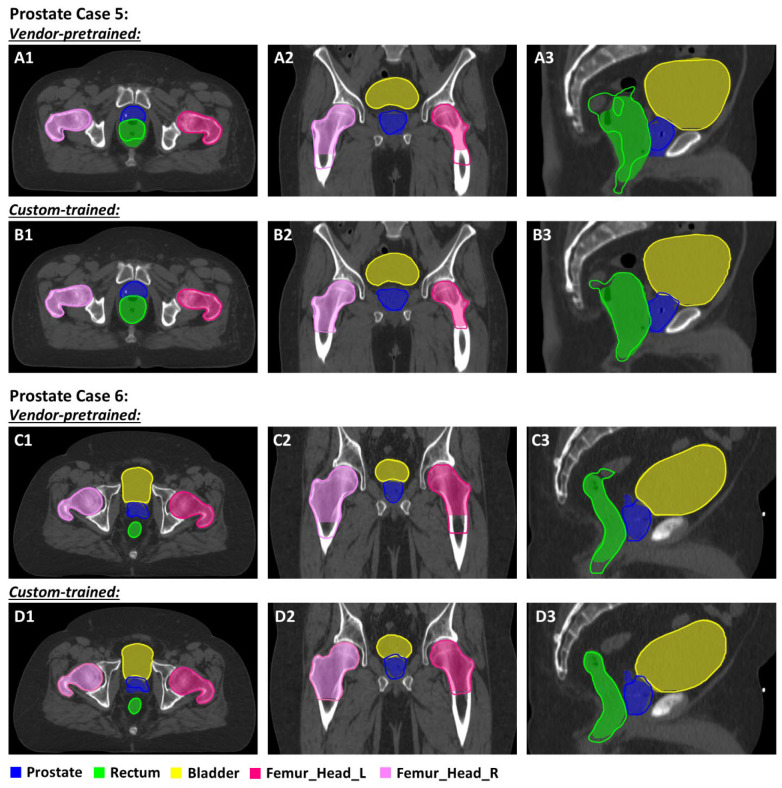
Comparison of contours derived from a vendor-pretrained prostate model (Case 5, (**A1**–**A3**); Case 6, (**C1**–**C3**)) and a custom-trained prostate model (**B1**–**B3**,**D1**–**D3**). Color wash indicates manually derived contours. Abbreviations: _L, left; _R, right; Femur_Head, femoral head.

**Figure 4:**
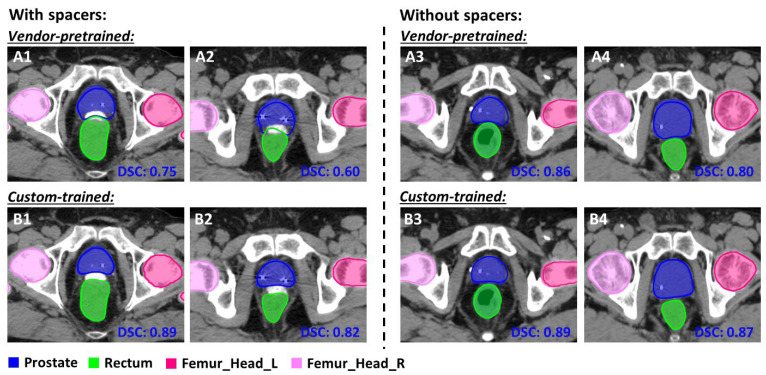
Comparison of auto-segmented prostates with and without a rectal spacer device. The Dice similarity coefficients (DSCs) for the prostate in each panel are shown in blue. (**A1**–**A4**) Auto-segmented contours from the vendor-pretrained prostate model. (**B1**–**B4**) Auto-segmented contours from the custom-trained prostate model. Color wash indicates clinically approved contours.

Auto-segmented contours for two patients with prostate cancer (Cases 5 and 6) are shown in Figure 3. In Case 5, the vendor-pretrained model under-delineated the inferior portion of the prostate [Figure 3 (Case 5-A3)]. In Case 6, neither model adequately delineated the prostate [Figure 3 (Case 6)]. The vendor-pretrained model generated contours for the anorectum that encompassed the combination of the anal canal and rectum, which are visible at the more inferior end in Figure 3 (Case 5-A3, Case 6-C3). A discrepancy was also observed at the superior end of the rectum. The custom-trained model contoured the femoral heads to align with the same level of inferior extension as the clinically approved contours, but the vendor-pretrained contours were more inclusive [Figure 3 (Case 5-A2,B2; Case 6-C2,D2)]. Notably, our institutional practice is to use spaceOAR hydrogel spacers (Boston Scientific, Houston, TX, USA) or Barrigel rectal spacers (Palette Life Sciences, Santa Barbara, CA, USA) for patients with prostate cancer to minimize the dose to the rectum (some of the patients in this study were treated prior to its adoption as a standard procedure). The performance of the vendor-pretrained model was significantly worse than the custom-trained model when a rectal spacer was present, as shown in Figure 4. The vendor-pretrained model misclassified the rectal spacer as part of the prostate or rectum [Figure 4(A1,A2)], probably because of a lack of exposure to this specific data during the training phase. In contrast, the custom-trained model accurately separated the rectal spacer from the prostate and rectum [Figure 4(B1,B2)]. In cases without a rectal spacer, the vendor-pretrained model performed better, as indicated by the higher DSC [Figure 4(A3,A4)]. Despite this improvement; however, the vendor-pretrained model performance was still inferior to that of the custom-trained model [Figure 4(B3,B4)].

### 3.4. Dosimetric Evaluation Results

**Figure 5:**
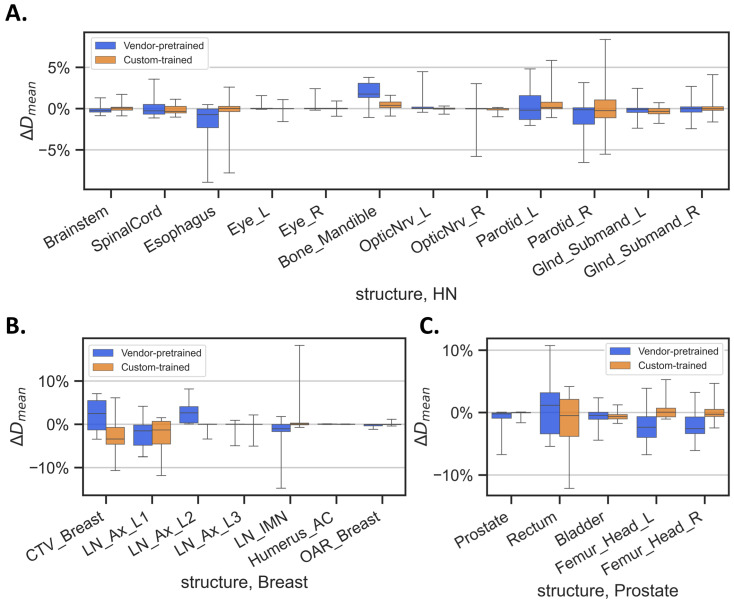
Errors in the mean dose (ΔD_mean_) delivered to each structure by the two models in: (**A**) head-and-neck (HN) cancer, (**B**) breast cancer, and (**C**) prostate cancer. ΔD_mean_ is defined as the difference between the mean dose represented by the auto-segmented contour and that of the clinically approved contour, normalized to the prescription dose. Abbreviations: _L, left; _R, right; OpticNrv, optic nerve; Glnd_Submand, submandibular gland; CTV_Breast, clinical target volume of breast; LN_Ax_L1- LN_Ax_L3, level 1–3 axillary lymph nodes; LN_IMN, internal mammary lymph nodes; Humerus_AC, humeral head and acromioclavicular joint.

**Figure 6:**
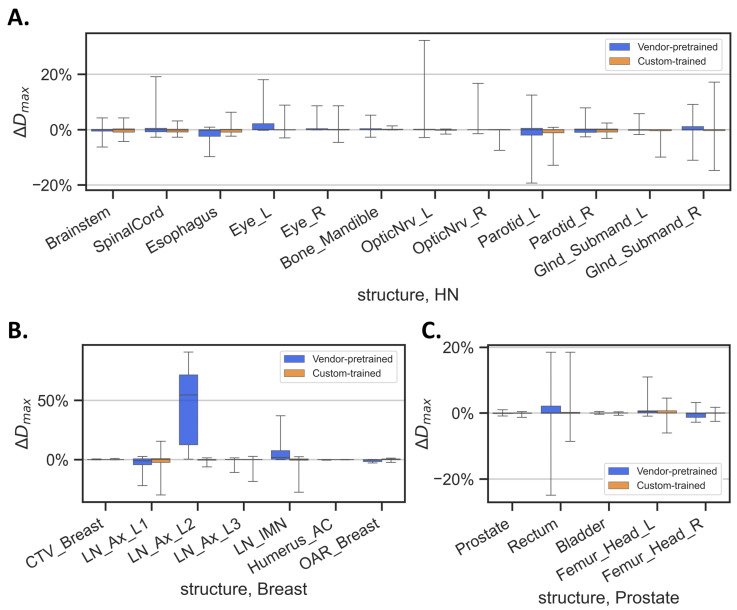
Errors in maximum dose (ΔD_max_) delivered to each structure by the two models in: (**A**) head-and-neck (HN) cancer, (**B**) breast cancer, and (**C**) prostate cancer. ΔD_max_ is defined as the difference between the maximum dose represented by the auto-segmented contour and that of the clinically approved contour, normalized to the prescription dose. Abbreviations: _L, left; _R, right; OpticNrv, optic nerve; Glnd_Submand, submandibular gland; CTV_Breast, clinical target volume of breast; LN_Ax_L1- LN_Ax_L3, level 1–3 axillary lymph nodes; LN_IMN, internal mammary lymph node; Humerus_AC, humeral head and acromioclavicular joint.

Finally, the errors in the mean dose and maximum dose calculated for the auto-segmented contours versus the clinically approved contours at the various anatomic sites within the HN, breast, and prostate structure categories are shown in Figure 5 and Figure 6. Details of these comparisons are provided in Supplementary Materials. On average, the custom-trained HN model led to better dosimetric estimates of ΔD_mean_ in the brainstem, esophagus, Bone_Mandible, and OpticNrv_L (Figure 5A), and more consistent dosimetric estimates of ΔD_max_ in the esophagus, Eye_L, and OpticNrv (Figure 6A) relative to the vendor-pretrained HN model. Notable discrepancies between breast models were seen in ΔD_mean_ in the LN_Ax_L2, Humerus_AC, and contralateral breast (Figure 5B), and in ΔD_max_ in the LN_Ax_L2 (Figure 6B). The significantly larger ΔD_mean_ and ΔD_max_ in vendor-pretained LN_Ax_L2 mainly came from the discrepancy in delineation preference as described beforehand. ΔD_mean_ estimates in the prostate and femoral heads were better from the custom-trained prostate model than those from the vendor-pretrained prostate model (Figure 5C)_._ The DVH curves for Cases 1 to 6 (shown in Figure 1, Figure 2 and Figure 3) are shown in Supplementary Materials.

## 4. Discussion

Contouring structures for radiation therapy are confounded by variability in data arising from adherence to diverse international or institutional guidelines, resulting in discrepancies in contour quality and consistency that are further compounded by inter- and intra-observer variability. Moreover, the existence of distinct treatment preferences and variations in patient populations and image acquisition protocols among institutions introduces additional complexities, further hindering the ability to attain consistent imaging results. Data variations in DL-based auto-segmentation algorithms underscore the need for meticulous QA procedures and potential manual corrections to ensure the reliability and accuracy of the algorithms. Consequently, the clinical acceptability of auto-segmented contours often becomes unsatisfactory, particularly when data variation is substantial.

The benefits of custom-trained commercial models come from two sources. First, training based on institutional data allows the incorporation of specific institutional guidelines and clinical practices. At most institutions, the development of clinically approved contours is informed by consensus guidance of published international guidelines and the collective expertise of clinical groups. Although some of these institution-specific preferences may not have significant clinical consequences, others, such as those pertaining to the parotid gland and rectum, would have considerable clinical impact. Nevertheless, ensuring that contouring is performed consistently, and dose-volume constraints are met during treatment planning remains crucial for delivering high-quality radiation therapy. When vendor-pretrained DL-based auto-segmentation algorithms are used, the need to manually edit the auto-segmented contours according to various preferences compromises the anticipated benefits of time- and labor-savings. Moreover, some institution-specific structures that are pertinent to clinical practice may not be encompassed within existing commercial DL-based models. For example, aggressive treatments for breast cancer may involve the delineation of extended lymph nodes, which are not typically included in vendor-pretrained commercial models. The findings reported here indicate that a relatively small amount of institutional data is adequate to train custom DL-based models from scratch, and that such models demonstrate performance comparable to vendor-pretrained models. This flexibility allows institutions to train specific models to auto-segment institution-specific structures.

A second benefit of using custom-trained models is enhanced robustness via ensuring homogenous training and testing data. One of the challenges encountered by DL-based auto-segmentation algorithms is diminished performance in the presence of heterogeneous data. For instance, in the case of prostate cancer, high-intensity rectal spacers are readily identified by the custom-trained model, but the vendor-pretrained model, having not been exposed to such data, fails to accurately identify the rectal spacer, resulting in low-quality contours. Variations in scanners and imaging protocols also introduce discrepancies in image quality, noise, contrast, and scan range, all of which significantly influence the predictive capacities of DL-based algorithms [25]. In some instances, the delineation of structures can be totally inaccurate because of differences in scan range. The use of a user-collected dataset that is consistent with and representative of routine clinical practice and patient population serves to enhance the robustness of DL-based predictions. Moreover, custom-trained models can be tailored for specific clinical scenarios. For example, after surgery for prostate cancer, delineation of the prostate bed or prostate fossa, which may not be successfully accomplished by a single prostate model, requires the separate curation of datasets and model training to improve the specificity of the segmentation tasks.

Commercial DL-based models are a beneficial resource for institutions that may lack clinical or technical resources. The well-established interface and optimization of cloud-based algorithms in particular have contributed to more efficient clinical workflows. Likewise, custom-trained models, developed and integrated within commercial toolkits, have processing properties that are comparable to their vendor-pretrained counterparts. The average prediction time for all custom-trained models in this study was less than 30 s for the preparation of all contours within the TPS. In contrast, our in-house models, although perhaps having superior performance, occasionally required more than 10 min to predict contours for a single patient. The technical requirements for training DL-based models have been alleviated through the use of custom-training toolkits, thereby enhancing the accessibility of customized DL-based algorithms. Therefore, custom-trained models integrated within the commercial toolkits can effectively harness the advantages of both in-house models and commercial vendor-pretrained models.

Our experiments further showed that a relatively small amount of data that adequately represent institutional preferences and patient population is sufficient to develop models that are on par with vendor-pretrained models. The inclusion of larger datasets may further improve model performance. However, given the substantial effort involved in curating large and representative datasets, a more generalized approach for the development of custom-trained models involves fine-tuning the vendor-pretrained models with institutional datasets through transfer learning. Several commercial vendors have introduced toolkits for DL-based transfer learning, which have shown considerable improvements in fine-tuned models, even with a limited number of training samples [43].

Our study has limitations, one of which is the absence of subjective evaluation by physicians. However, our primary aim was to provide insights into the benefits associated with custom-trained models that use vendor-supplied DL-based toolkits. Our comprehensive qualitative and quantitative evaluations revealed that training with a relatively small institutional dataset is sufficient to develop custom-trained models that perform comparably across most structures yet significantly outperform vendor-pretrained models in the case of institution-specific guidelines for specific structures. Another limitation was the lack of a comprehensive assessment of the optimal number of patients required for custom training. Although we acknowledge that the use of selected data can significantly influence the assessment of model performance, conducting such analyses could provide benchmark values for evaluation metrics, thereby offering valuable insights into the acceptability of custom-trained models.

In this study, clinical contours used for model training were generated following institutional consensus guidelines, ensuring consistent quality. This rigorous data curation enhanced the performance of custom-trained models. However, variability in manually delineated contours could compromise model accuracy with limited data, potentially offsetting the benefits of custom training. Therefore, a thorough assessment of training data quality remains essential. Another potential limitation of custom-trained models is reduced generalizability; models trained on highly consistent institutional data may underperform in cases involving uncommon anatomical variations. Thus, implementing comprehensive QA procedures is critical before deploying custom-trained models in clinical practice. This study presents a workflow for comparing custom-trained and vendor-pretrained models, enabling the identification of model reliability and potential pitfalls regarding specific structures.

## 5. Conclusions

We demonstrated the effectiveness of custom-trained, DL-based auto-segmentation models integrated within vendor-supplied toolkits in terms of accommodating institutional preferences and imaging protocols. Our results indicate that a relatively small dataset (approximately 30 samples) could be adequate for developing custom models from scratch while simultaneously yielding performance that is comparable or superior to that of commercial vendor-pretrained counterparts, depending on institutional preferences with regard to specific structures. This study provides valuable insights into the implications of data variation and local clinical practices during the implementation of DL-based auto-segmentation. Nevertheless, meticulous data curation and rigorous QA processes remain essential to ensure the reliability and clinical applicability of these automated tools.

## Figures and Tables

**Table 1:** Details of contour guidelines and proportions of training/validation data in custom-trained and vendor-pretrained segmentation models. Abbreviations: HN, head and neck; LN_IMN, internal mammary lymph node; RTOG, Radiation Therapy Oncology Group.

Model	References for Contouring Guidelines	Localization Submodel		Segmentation Submodel	
		Training	Validation	Training	Validation
Vendor-pretrained HN	Brouwer et al. [31]	85 *	14	Ranging from 80 (brainstem) to 170 (eye)	Ranging from 14 (brainstem) to 30 (eye)
Custom-trained HN	Brouwer et al. [31] Mir et al. [32] Scoccianti et al. [33] Eekers et al. [34] Van De Water et al. [35]	30	5	30	5
Vendor-pretrained Breast	Mir et al. [32] Offersen et al. [36]	141 *	24	Ranging from 97 (LN_IMN) to 146 (breast)	Ranging from 17 (LN_IMN) to 24 (breast)
Custom-trained Breast	RTOG breast atlases [37]	34 (left); 39 (left)	5 (left); 5(left)	34 (left); 39 (left)	5 (left); 5(left)
Vendor-pretrained Prostate	Mir et al. [32] Gay et al. [38] Nestle et al. [39] Nyholm et al. [40]	unknown **	unknown **	unknown **	unknown **
Custom-trained Prostate	Mir et al. [32] Gay et al. [38] Nestle et al. [39] Michalski et al. [41] Hall et al. [42]	30	5	30	5

* Extra negative CT samples of other scan regions were also used in the vendor-pretrained localization submodel but were not counted here. ** Vendor did not provide this information.

**Table 2:** Metrics indicating similarity between auto-segmented contours and clinically approved contours of diverse anatomic sites. Values are mean ± standard deviation. The effect size is calculated using Cohen’s d comparing custom-trained vs. vendor-pretrained models. Listed *p* values are from two-sided Wilcoxon signed-rank tests comparing DSCs and MSDs from custom-trained and vendor-pretrained models for each structure. Bold indicates custom-trained model achieves better conformity with clinically approved contours with a *p* value less than 0.05. The symbol * indicates custom-trained model performed inferiorly in the structure than vendor-pretrained model. Abbreviations: _L, left; _R, right; OpticNrv, optic nerve; Glnd_Submand, submandibular gland; CTV_Breast, clinical target volume of breast; LN_Ax_L1- LN_Ax_L3, level 1–3 axillary lymph nodes; LN_IMN, internal mammary lymph node; Humerus_AC, humeral head and acromioclavicular joint; Femur_Head, femoral head.

Site	Structures		Dice Similarity Coefficient (DSC)				Mean Surface Distance (MSD), mm		
		Vendor-Pretrained	Custom-Trained	Effect Size	*p* Value	Vendor-Pretrained	Custom-Trained	Effect Size	*p* Value
**Head and neck (*n* = 18)**	Overall	0.81 ± 0.12	**0.86 ± 0.11**	0.19	<0.001	0.81 ± 0.56	**0.77 ± 0.76**	−0.26	<0.001
	Brainstem	0.89 ± 0.03	**0.91 ± 0.02**	0.77	0.021	1.11 ± 0.48	0.90 ± 0.35	−0.48	0.119
	Spinal Cord	0.88 ± 0.03	0.88 ± 0.03	−0.13 *	0.442	0.49 ± 0.15	0.45 ± 0.11	−0.29	0.417
	Esophagus	0.83 ± 0.05	0.86 ± 0.07	0.41	0.093	0.91 ± 0.48	0.87 ± 0.77	−0.06	0.464
	Eye_L	0.92 ± 0.02	0.92 ± 0.02	0.23	0.393	0.47 ± 0.21	0.40 ± 0.17	−0.35	0.119
	Eye_R	0.93 ± 0.02	0.93 ± 0.02	0.00	0.865	0.40 ± 0.16	0.38 ± 0.15	−0.10	0.495
	Bone_Mandible	0.89 ± 0.02	**0.92 ± 0.03**	1.39	0.002	1.01 ± 0.23	**0.60 ± 0.80**	−0.67	0.002
	OpticNrv_L	0.64 ± 0.06	0.69 ± 0.08	0.39	0.339	1.15 ± 0.60	0.70 ± 0.24	−1.00	0.064
	OpticNrv_R	0.66 ± 0.07	0.74 ± 0.05	0.99	0.054	1.08 ± 0.47	**0.59 ± 0.19**	−1.23	0.014
	Parotid_L	0.86 ± 0.04	**0.90 ± 0.04**	01.00	˂0.001	1.32 ± 0.68	**0.86 ± 0.43**	−0.78	˂0.001
	Parotid_R	0.87 ± 0.03	**0.89 ± 0.05**	0.68	0.004	1.14 ± 0.41	0.96 ± 0.48	−0.39	0.074
	Glnd_Submand_L	0.80 ± 0.12	0.77 ± 0.22	−0.02 *	0.296	1.02 ± 0.59	1.44 ± 1.87	0.21 *	0.326
	Glnd_Submand_R	0.80 ± 0.16	0.82 ± 0.16	0.08	0.298	1.05 ± 0.94	1.00 ± 0.96	−0.05	0.495
**Breast (*n* = 21)**	Overall	0.67 ± 0.16	**0.80 ± 0.11**	0.92	˂0.001	4.85 ± 2.44	**2.42 ± 1.49**	−1.21	˂0.001
	CTV_Breast	0.80 ± 0.05	**0.88 ± 0.06**	1.35	˂0.001	5.26 ± 1.62	**3.44 ± 2.22**	−0.93	˂0.001
	LN_Ax_L1	0.76 ± 0.05	**0.80 ± 0.05**	0.78	0.002	3.95 ± 1.39	**3.01 ± 1.24**	−0.69	0.002
	LN_Ax_L2	0.39 ± 0.07	**0.79 ± 0.06**	5.81	˂0.001	8.24 ± 3.16	**1.75 ± 0.64**	−2.78	˂0.001
	LN_Ax_L3	0.69 ± 0.12	0.68 ± 0.08	−0.03 *	0.812	2.06 ± 0.82	2.19 ± 1.09	0.14 *	0.759
	LN_IMN	0.56 ± 0.09	**0.68 ± 0.08**	1.39	˂0.001	4.55 ± 1.02	**1.45 ± 1.17**	−2.85	˂0.001
	Humerus_AC	0.66 ± 0.01	**0.83 ± 0.09**	1.67	˂0.001	5.55 ± 1.60	**2.22 ± 1.40**	−2.16	˂0.001
	OAR_Breast	0.83 ± 0.04	**0.90 ± 0.03**	1.95	˂0.001	4.33 ± 1.03	**2.85 ± 0.99**	−1.43	˂0.001
**Prostate (*n* = 18)**	Overall	0.87 ± 0.09	**0.92 ± 0.06**	0.68	<0.001	2.17 ± 1.39	**1.21 ± 1.00**	−0.84	<0.001
	Prostate	0.77 ± 0.08	**0.86 ± 0.06**	1.17	<0.001	2.79 ± 1.12	**1.64 ± 0.78**	−1.16	0.001
	Rectum	0.81 ± 0.07	**0.88 ± 0.06**	0.97	0.007	2.85 ± 1.68	**1.66 ± 0.99**	−0.84	0.024
	Bladder	0.96 ± 0.02	0.96 ± 0.02	0.28	0.486	0.96 ± 0.92	0.76 ± 0.41	−0.37	0.153
	Femur_Head_L	0.90 ± 0.04	**0.95 ± 0.03**	1.31	0.001	2.19 ± 1.16	**0.97 ± 1.19**	−1.01	0.002
	Femur_Head_R	0.91 ± 0.03	**0.95 ± 0.03**	1.34	0.001	2.07 ± 1.03	**0.96 ± 1.01**	−1.06	0.005

## Data Availability

The data can be made available upon reasonable request to Jinzhong Yang.

## Supplementary Materials

The following supporting information can be downloaded at: https://www.mdpi.com/article/10.3390/diagnostics14242851/s1, Figure S1: Dice similarity coefficients (DSCs) of each structure by the two models in: (A) head-and-neck (HN) cancer, (B) breast cancer, and (C) prostate cancer. Figure S2: Mean surface distances (MSDs, in mm) of each structure by the two models: (A) head-and-neck (HN) cancer, (B) breast cancer, and (C) prostate cancer. Table S1: Normalized error in mean dose (ΔD_mean_) and error in maximum dose (ΔD_max_) calculated for auto-segmented contours and manual contours. Table S2: Error in the mean dose (ΔD_mean_) and error in the maximum dose (ΔDmax) calculated for auto-segmented contours and manual contours. Figure S3: Dose-volume histograms (DVHs) of structures from a head-and-neck cancer clinical plan (Case 1, see Figure 1), calculated based on manual contours (solid lines) and auto-segmented contours (dotted lines) generated by (A) a vendor-pretrained model and (B) a custom-trained model. Figure S4: Dose-volume histograms (DVHs) of structures from a head-and-neck clinical plan (Case 2, see Figure 1), calculated based on manual contours (solid lines) and auto-segmented contours (dotted lines) generated by (A) a vendor-pretrained model and (B) a custom-trained model. Figure S5: Dose-volume histograms (DVHs) of structures from a breast cancer clinical plan (Case 3, see Figure 2) calculated based on manual contours (solid lines) and auto-segmented contours (dotted lines) generated by (A) a vendor-pretrained model and (B) a custom-trained model. Figure S6: Dose-volume histograms (DVHs) of structures from a breast cancer clinical plan (Case 4, see Figure 2) calculated based on manual contours (solid lines) and auto-segmented contours (dotted lines) generated by (A) a vendor-pretrained model and (B) a custom-trained model. Figure S7: Dose-volume histograms (DVHs) of structures from a prostate cancer clinical plan (Case 5, see Figure 3) calculated based on manual contours (solid lines) and auto-segmented contours (dotted lines) generated by (A) a vendor-pretrained model and (B) a custom-trained model. Figure S8: Dose-volume histograms (DVHs) of structures from a prostate cancer clinical plan (Case 6, see Figure 3) calculated based on manual contours (solid lines) and auto-segmented contours (dotted lines) generated by (A) a vendor-pretrained model and (B) a custom-trained model.
