# Supplementary material for: Comparison of Vendor-Pretrained and Custom-Trained Deep Learning Segmentation Models for Head-and-Neck, Breast, and Prostate Cancers
Source: Diagnostics (Basel). 2024 Dec 18;14(24):2851. doi: 10.3390/diagnostics14242851 (PMC11675285; doi:10.3390/diagnostics14242851)
Supplement: Supplementary file 1 [file diagnostics-14-02851-s001.zip › diagnostics-3355381-supplementary.pdf]

**Geometric evaluation**

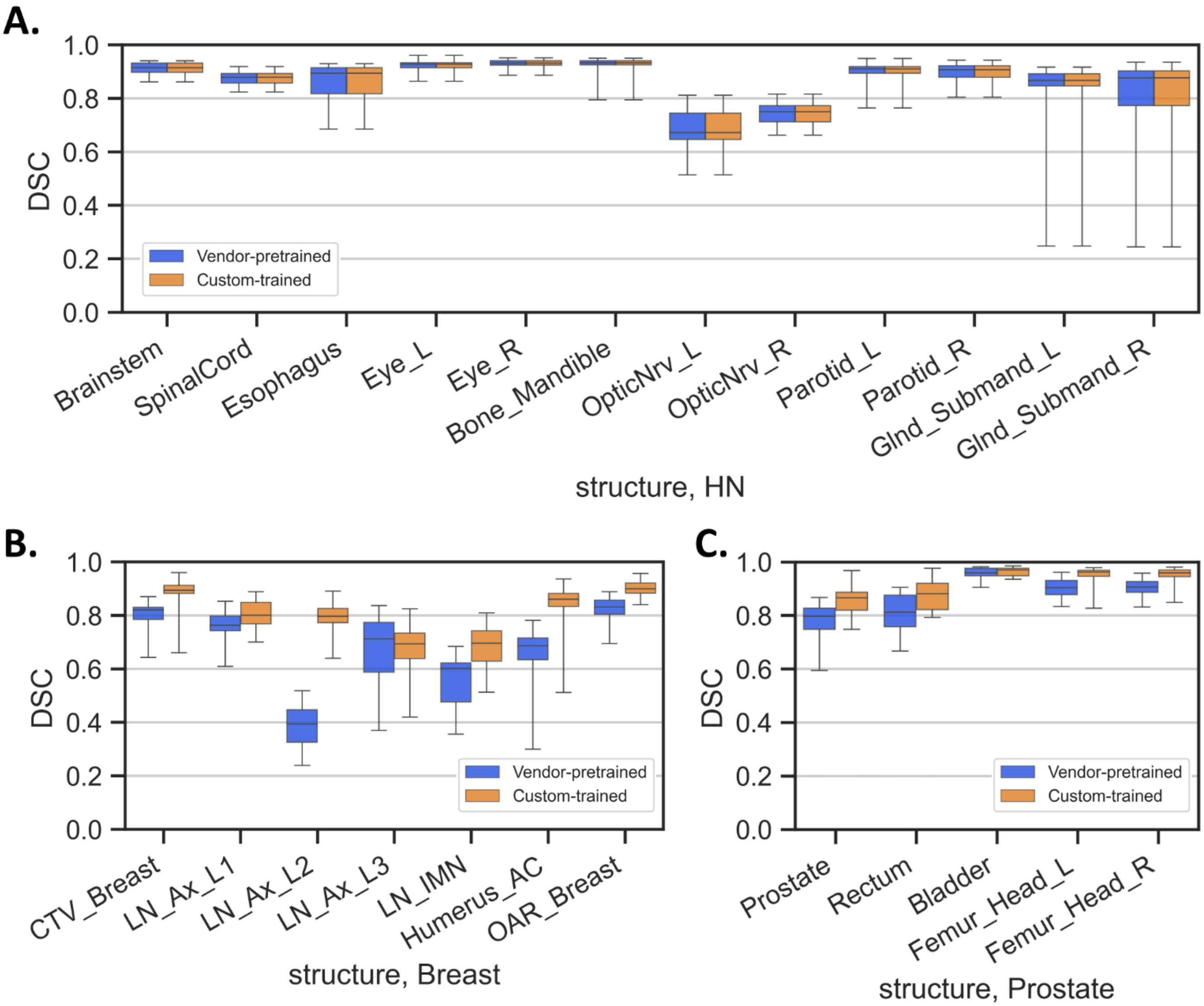


**Supplementary Figure S1**. Dice similarity coefficients (DSCs) of each structure by the two models in: (A) head-and-neck (HN) cancer, (B) breast cancer, and (C) prostate cancer. Abbreviations: _L, left; _R, right; OpticNrv, optic nerve; Glnd_Submand, submandibular gland; CTV_Breast, clinical target volume of breast; LN_Ax_L1- LN_Ax_L3, level 1-3 axillary lymph nodes; LN_IMN, internal mammary lymph nodes; Humerus_AC, humeral head and acromioclavicular joint.

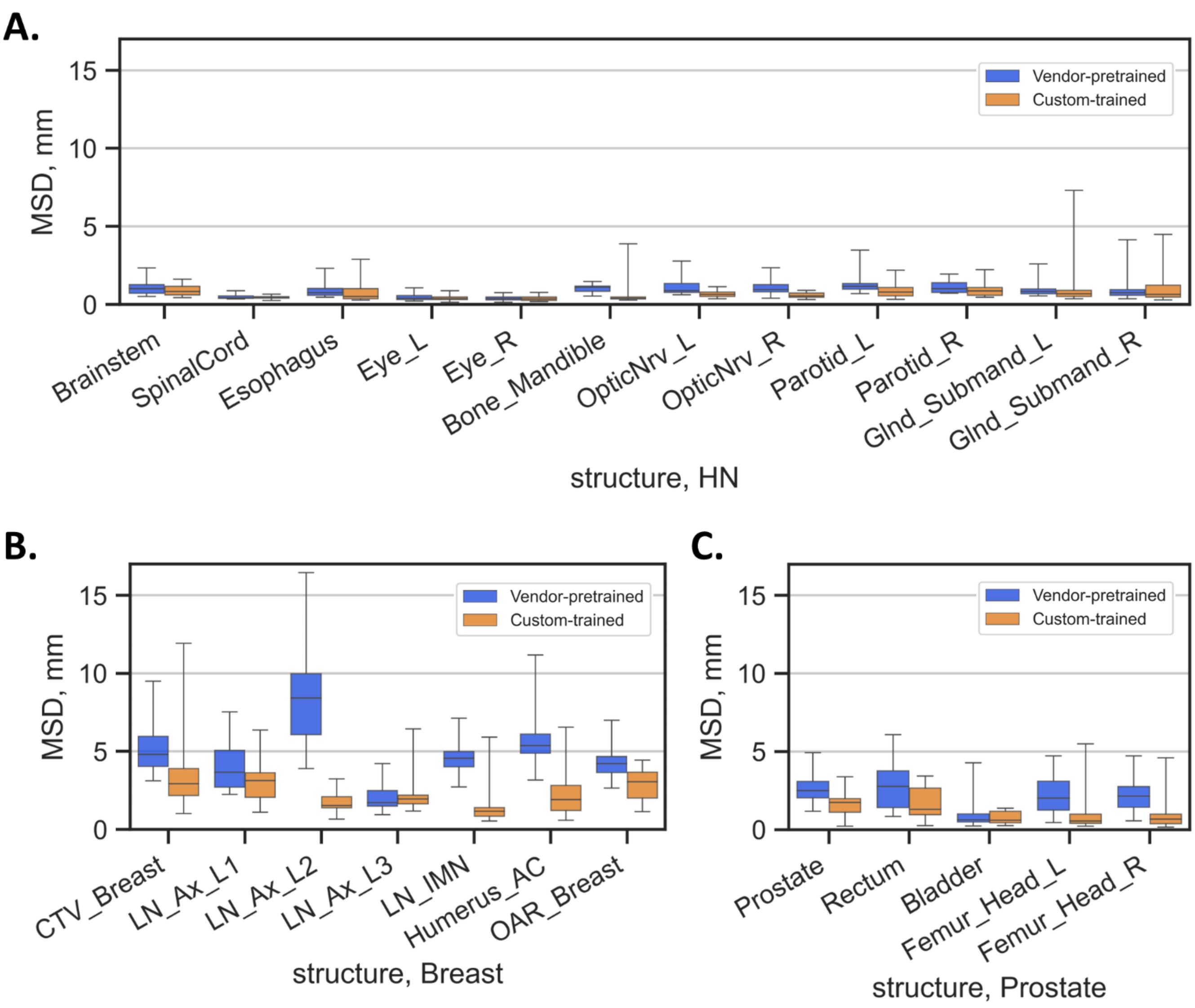


**Supplementary Figure S2**. Mean surface distances (MSDs, in mm) of each structure by the two models in: (A) head-and-neck (HN) cancer, (B) breast cancer, and (C) prostate cancer. Abbreviations: _L, left; _R, right; OpticNrv, optic nerve; Glnd_Submand, submandibular gland; CTV_Breast, clinical target volume of breast; LN_Ax_L1- LN_Ax_L3, level 1-3 axillary lymph nodes; LN_IMN, internal mammary lymph nodes; Humerus_AC, humeral head and acromioclavicular joint.

## Dosimetric Evaluation

**Supplementary Table S1**. Normalized error in mean dose ($\Delta D_{mean}$) and error in maximum dose ($\Delta D_{max}$) calculated for auto-segmented contours and manual contours.

| Site | Structures | $\Delta D_{mean}$, normalized to prescription dose | | | $\Delta D_{max}$, normalized to prescription dose | | |
|---|---|---|---|---|---|---|---|
| | | Vendor-Pretrained Model | Custom-Trained Model | *P* Value* | Vendor-Pretrained Model | Custom-Trained Model | *P* Value* |
| **Head and neck** | Brainstem | -0.15% (0.52%) | 0.15% (0.66%) | 0.005 | -0.38% (2.49%) | -0.42% (2.03%) | 0.328 |
| | Spinal Cord | -0.03% (1.08%) | -0.13% (0.61%) | 0.323 | 0.94% (4.69%) | -0.30% (1.45%) | 0.508 |
| | Esophagus | -1.56% (2.18%) | -0.40% (2.21%) | 0.003 | -1.54% (2.45%) | 0.38% (2.38%) | 0.041 |
| | Eye_L | 0.24% (0.52%) | -0.05% (0.53%) | 0.074 | 2.36% (4.75%) | 0.26% (2.34%) | 0.003 |
| | Eye_R | 0.23% (0.67%) | -0.03% (0.38%) | 0.562 | 0.67% (2.01%) | 0.31% (2.43%) | 0.221 |
| | Bone_Mandible | 1.73% (1.48%) | 0.46% (0.70%) | 0.000 | 0.21% (1.45%) | 0.19% (0.37%) | 0.214 |
| | OpticNrv_L | 0.40% (1.09%) | -0.04% (0.23%) | 0.002 | 1.94% (7.68%) | -0.32% (0.62%) | 0.025 |
| | OpticNrv_R | -0.20% (1.67%) | -0.15% (0.32%) | 0.074 | 0.99% (3.98%) | -0.84% (2.33%) | 0.123 |
| | Parotid_L | 0.29% (1.88%) | 0.63% (1.50%) | 0.782 | -1.49% (6.22%) | -2.09% (4.13%) | 0.975 |
| | Parotid_R | -0.70% (2.12%) | -0.01% (2.99%) | 0.900 | 0.21% (2.27%) | -0.23% (1.25%) | 0.814 |
| | Glnd_Submand_L | -0.07% (1.07%) | -0.34% (0.62%) | 0.677 | 0.43% (1.79%) | -0.98% (2.60%) | 0.612 |
| | Glnd_Submand_R | -0.08% (1.01%) | 0.19% (1.21%) | 0.670 | 0.62% (4.34%) | 0.48% (5.94%) | 0.327 |
| **Breast** | CTV_Breast | 2.14% (3.70%) | -2.59% (4.22%) | 0.004 | 0.03% (0.19%) | 0.08% (0.33%) | 0.317 |
| | LN_Ax_L1 | -2.21% (3.44%) | -2.84% (4.26%) | 0.375 | -3.55% (6.93%) | -1.79% (10.64%) | 0.922 |
| | LN_Ax_L2 | 2.65% (2.46%) | -0.35% (1.03%) | 0.002 | 45.57% (32.84%) | -0.55% (2.01%) | 0.002 |
| | LN_Ax_L3 | -0.41% (1.55%) | -0.29% (1.71%) | 0.999 | -0.91% (3.36%) | -1.63% (5.72%) | 0.173 |
| | LN_IMN | -2.13% (4.38%) | 2.10% (5.48%) | 0.064 | 7.23% (11.21%) | -3.08% (8.63%) | 0.008 |
| | Humerus_AC | 0.03% (0.04%) | 0.01% (0.03%) | 0.232 | -0.08% (0.14%) | -0.04% (0.09%) | 0.012 |
| | OAR_Breast | -0.30% (0.38%) | 0.03% (0.40%) | 0.002 | -0.89% (1.04%) | 0.03% (0.93%) | 0.008 |
| **Prostate** | Prostate | -1.12% (1.89%) | -0.12% (0.39%) | <0.001 | -0.02% (0.38%) | -0.09% (0.36%) | 0.347 |
| | Rectum | 0.95% (4.27%) | -1.04% (4.09%) | 0.054 | 0.65% (7.58%) | 0.93% (5.04%) | 0.249 |
| | Bladder | -0.61% (1.39%) | -0.58% (0.75%) | 0.068 | 0.00% (0.18%) | -0.07% (0.25%) | 0.401 |
| | Femur_Head_L | -2.10% (2.72%) | 0.48% (1.60%) | <0.001 | 1.22% (2.58%) | 0.18% (2.07%) | 0.064 |
| | Femur_Head_R | -2.03% (2.66%) | 0.19% (1.60%) | 0.001 | -0.37% (1.35%) | -0.20% (1.00%) | 0.331 |

Abbreviations: _L, left; _R, right; OpticNrv, optic nerve; Glnd_Submand, submandibular gland; CTV_Breast, clinical target volume of breast; LN_Ax_L1-LN_Ax_L3, level 1-3 axillary lymph nodes; LN_IMN, internal mammary lymph node; Humerus_AC, humeral head and acromioclavicular joint; Femur_Head, femoral head.
**P* from Wilcoxon signed rank test comparing custom-trained and vendor-pretrained models for each structure. Values in parentheses are standard deviations.

**Supplementary Table S2**. Error in mean dose ($\Delta D_{mean}$) and error in maximum dose ($\Delta D_{max}$) calculated for auto-segmented contours and manual contours.

| Site | Structures | $\Delta D_{mean}$, Gy | | | $\Delta D_{max}$, Gy | | |
|---|---|---|---|---|---|---|---|
| | | Vendor-Pretrained Model | Custom-Trained Model | *P* Value* | Vendor-Pretrained Model | Custom-Trained Model | *P* Value* |
| **Head and neck** | Brainstem | -0.12 (0.28) | 0.08 (0.43) | 0.005 | -0.25 (1.60) | -0.29 (1.31) | 0.213 |
| | Spinal Cord | -0.14 (0.37) | -0.10 (0.37) | 0.274 | 0.64 (3.22) | -0.21 (0.70) | 0.333 |
| | Esophagus | -0.71 (0.78) | -0.09 (0.85) | 0.003 | -0.99 (1.52) | 0.11 (1.30) | 0.041 |
| | Eye_L | 0.13 (0.29) | -0.03 (0.35) | 0.058 | 1.43 (3.07) | 0.18 (1.49) | 0.003 |
| | Eye_R | 0.10 (0.29) | -0.00 (0.22) | 0.495 | 0.41 (1.28) | 0.21 (1.53) | 0.173 |
| | Bone_Mandible | 1.04 (0.91) | 0.31 (0.41) | 0.001 | 0.09 (0.40) | 0.08 (0.14) | 0.214 |
| | OpticNrv_L | 0.16 (0.39) | -0.04 (0.14) | 0.002 | 0.59 (2.39) | -0.15 (0.33) | 0.025 |
| | OpticNrv_R | -0.13 (1.03) | -0.09 (0.22) | 0.129 | 0.68 (2.76) | -0.58 (1.62) | 0.123 |
| | Parotid_L | 0.10 (1.03) | 0.38 (0.91) | 0.782 | -0.65 (3.64) | -1.18 (2.50) | 0.925 |
| | Parotid_R | -0.40 (1.32) | -0.12 (1.48) | 0.782 | 0.00 (0.72) | -0.12 (0.57) | 0.754 |
| | Glnd_Submand_L | -0.06 (0.58) | -0.23 (0.41) | 0.622 | 0.18 (1.04) | -0.60 (1.56) | 0.612 |
| | Glnd_Submand_R | -0.04 (0.33) | 0.08 (0.77) | 0.583 | 0.16 (2.45) | 0.21 (3.72) | 0.484 |
| **Breast** | CTV_Breast | 0.67 (1.30) | -0.91 (1.64) | 0.004 | 0.01 (0.07) | 0.03 (0.12) | 0.317 |
| | LN_Ax_L1 | -0.65 (1.17) | -0.85 (1.18) | 0.432 | -1.22 (2.44) | -0.65 (3.72) | 0.999 |
| | LN_Ax_L2 | 0.86 (0.76) | -0.14 (0.41) | 0.002 | 14.62 (10.54) | -0.25 (0.78) | 0.002 |
| | LN_Ax_L3 | -0.17 (0.61) | -0.15 (0.65) | 0.999 | -0.39 (1.33) | -0.69 (2.26) | 0.214 |
| | LN_IMN | -0.73 (1.55) | 0.83 (2.19) | 0.064 | 2.56 (4.34) | -0.73 (2.29) | 0.008 |
| | Humerus_AC | 0.01 (0.02) | 0.00 (0.01) | 0.322 | -0.03 (0.06) | -0.02 (0.03) | 0.012 |
| | OAR_Breast | -0.11 (0.14) | 0.00 (0.15) | 0.002 | -0.32 (0.39) | 0.00 (0.32) | 0.008 |
| **Prostate** | Prostate | -0.57 (0.93) | -0.06 (0.21) | <0.001 | 0.01 (0.23) | -0.04 (0.19) | 0.347 |
| | Rectum | 0.42 (2.29) | -0.65 (2.22) | 0.048 | -0.08 (4.56) | 0.24 (2.41) | 0.249 |
| | Bladder | -0.36 (0.81) | -0.32 (0.48) | 0.078 | 0.00 (0.07) | -0.04 (0.13) | 0.327 |
| | Femur_Head_L | -1.26 (1.50) | 0.21 (0.70) | <0.001 | 0.56 (1.15) | 0.07 (1.05) | 0.055 |
| | Femur_Head_R | -1.24 (1.49) | 0.05 (0.74) | 0.001 | -0.21 (0.72) | -0.14 (0.53) | 0.397 |

Abbreviations: _L, left; _R, right; OpticNrv, optic nerve; Glnd_Submand, submandibular gland; CTV_Breast, clinical target volume of breast; LN_Ax_L1-LN_Ax_L3, level 1-3 axillary lymph nodes; LN_IMN, internal mammary lymph node; Humerus_AC, humeral head and acromioclavicular joint; Femur_Head, femoral head.

**P* from Wilcoxon signed rank test comparing custom-trained and vendor-pretrained models for each structure. Values in parentheses are standard deviations.

**Dose-Volume Histograms for Cases 1-6 (as shown in Main Text Figures 1-3)**

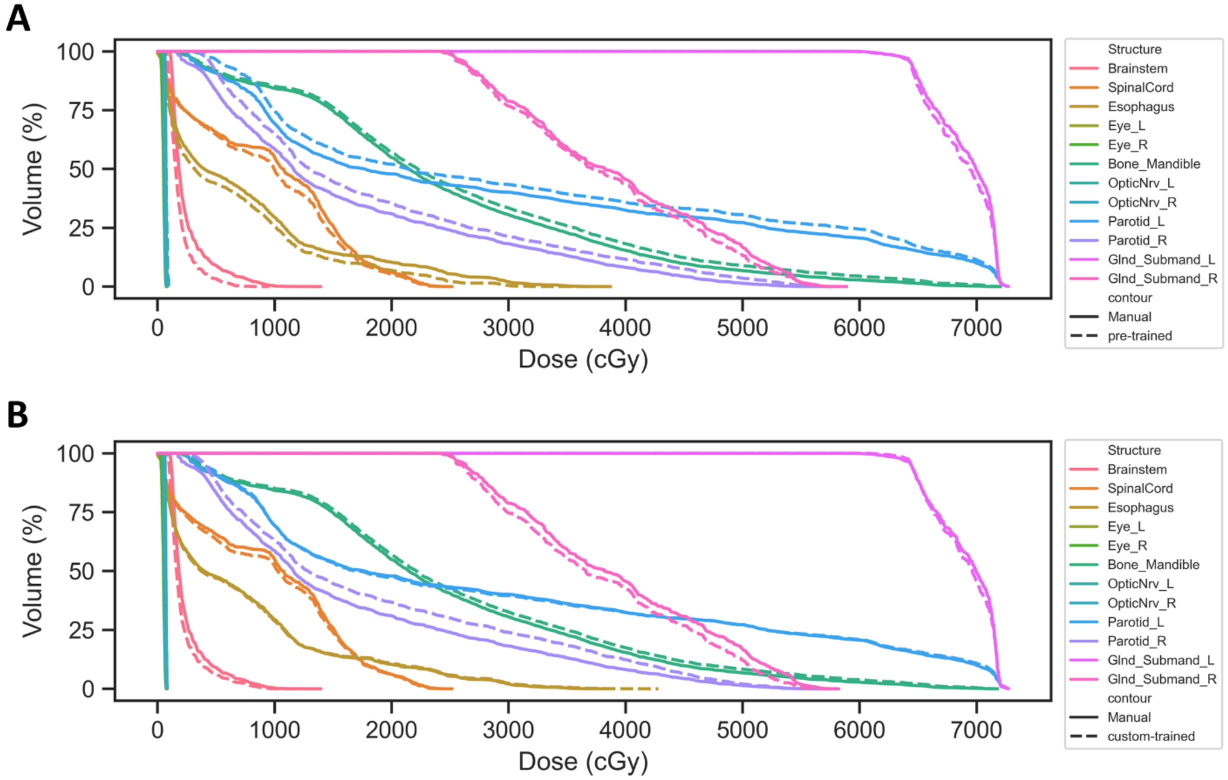


**Supplementary Figure S3**. Dose-volume histograms (DVHs) of structures from a head-and-neck cancer clinical plan (Case 1, see Figure 1), calculated based on manual contours (solid lines) and auto-segmented contours (dotted lines) generated by (A) a vendor-pretrained model and (B) a custom-trained model. The custom-trained model showed more consistent DVHs for Brainstem, SpinalCord, Esophagus, Parotid_L and Glnd_Submand_L. Abbreviations: _L, left; _R, right; OpticNrv, optic nerve; Glnd_Submand, submandibular gland.

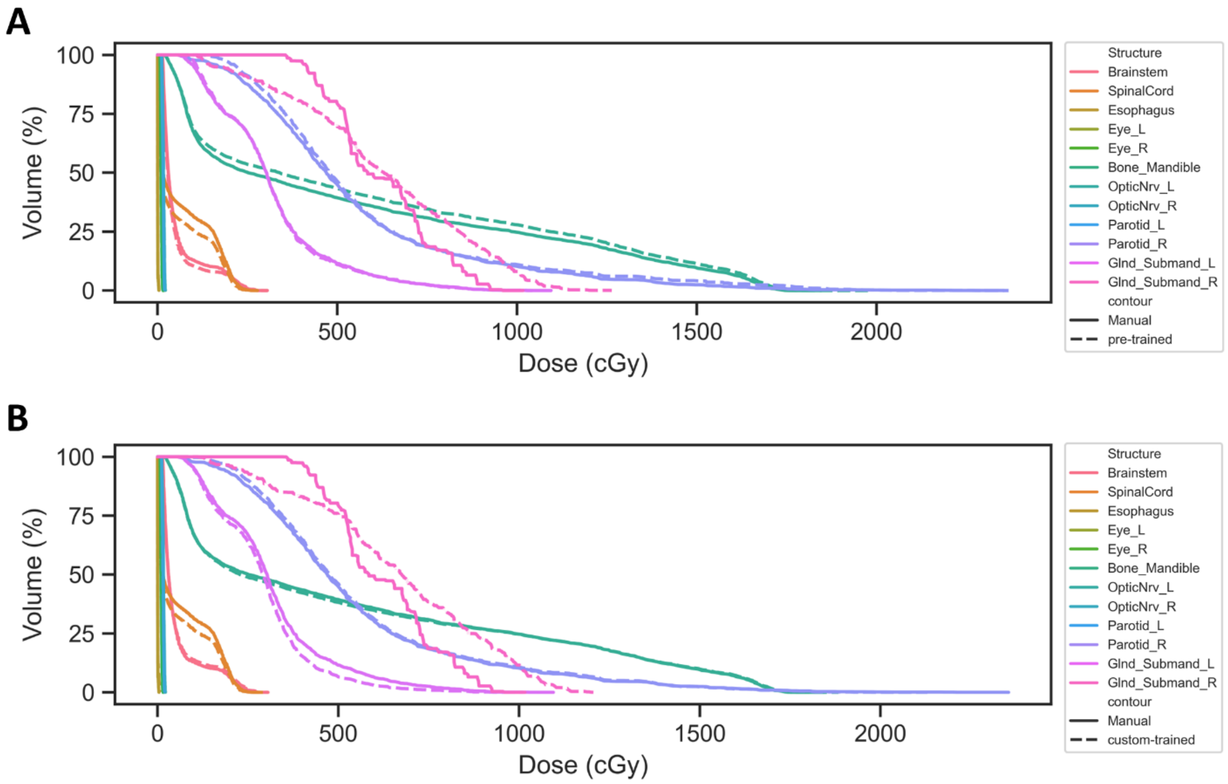


**Supplementary Figure S4**. Dose-volume histograms (DVHs) of structures from a head-and-neck clinical plan (Case 2, see Figure 1), calculated based on manual contours (solid lines) and auto-segmented contours (dotted lines) generated by (A) a vendor-pretrained model and (B) a custom-trained model. The custom-trained model showed more consistent DVHs for Brainstem, esophagus, and Bone_Mandible, but performed poorly for Glnd_Submand_R. Abbreviations: _L, left; _R, right; OpticNrv, optic nerve; Glnd_Submand, submandibular gland.

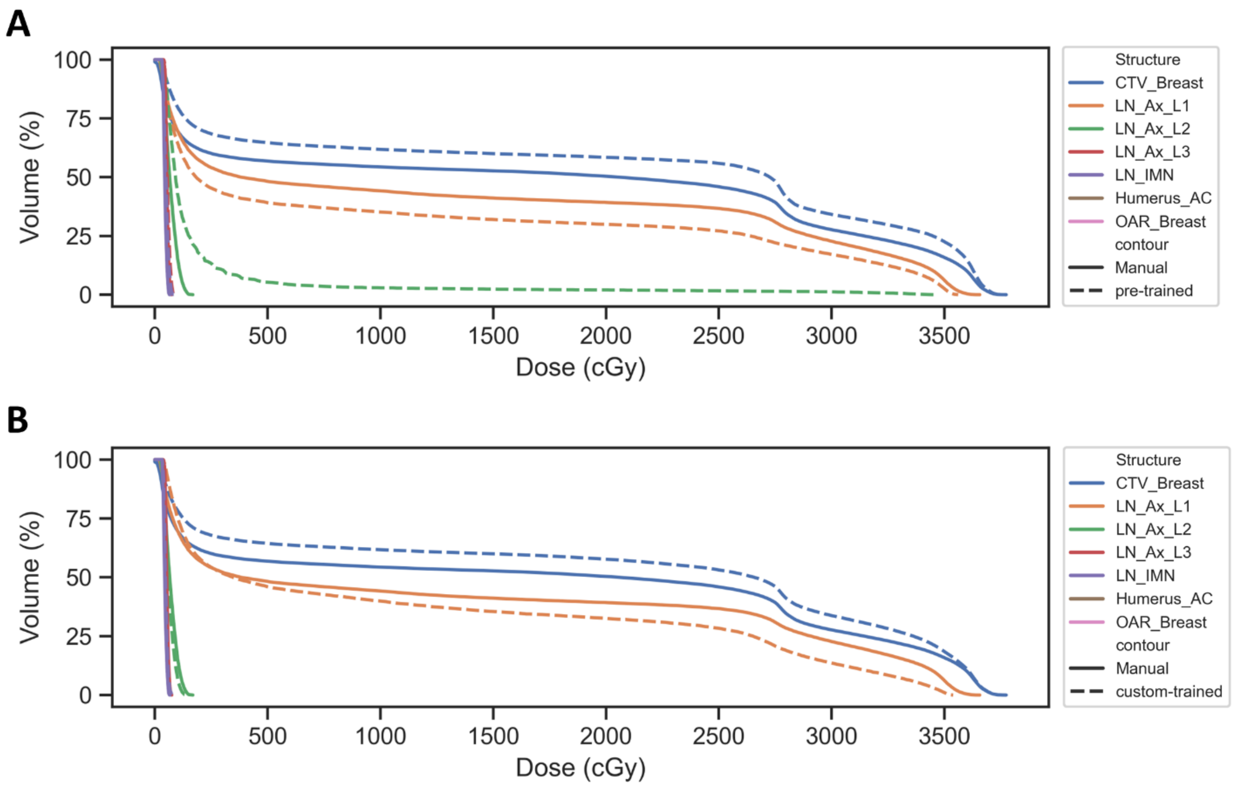


**Supplementary Figure S5**. Dose-volume histograms (DVHs) of structures from a breast cancer clinical plan (Case 3, see Figure 2) calculated based on manual contours (solid lines) and auto-segmented contours (dotted lines) generated by (A) a vendor-pretrained model and (B) a custom-trained model. Both models showed discrepancies in CTV_Breast and LN_Ax_L1, with significant inconsistencies in DVH in the LN_Ax_L2 generated by the vendor-pretrained model. Abbreviations: CTV_Breast, clinical target volume of breast; LN_Ax_L1- LN_Ax_L3, level 1 to level 3 axillary lymph nodes; LN_IMN, internal mammary lymph node; Humerus_AC, humeral head and acromioclavicular joint.

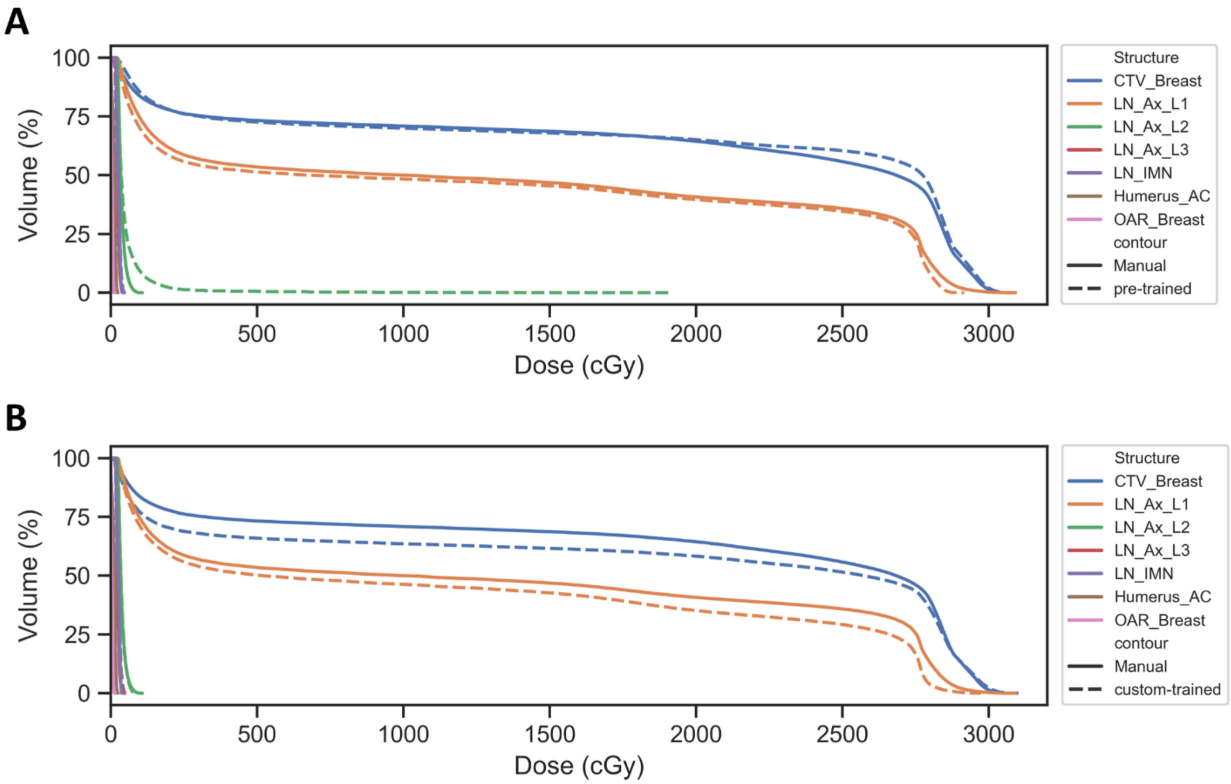


**Supplementary Figure S6**. Dose-volume histograms (DVHs) of structures from a breast cancer clinical plan (Case 4, see Figure 2) calculated based on manual contours (solid lines) and auto-segmented contours (dotted lines) generated by (A) a vendor-pretrained model and (B) a custom-trained model. The custom-trained model performed badly in both CTV_Breast and LN_Ax_L1, with significant inconsistencies in DVH in the LN_Ax_L2 generated by the vendor-pretrained model. Abbreviations: CTV_Breast, clinical target volume of breast; LN_Ax_L1- LN_Ax_L3, level 1 to level 3 axillary lymph nodes; LN_IMN, internal mammary lymph node; Humerus_AC, humeral head and acromioclavicular joint.

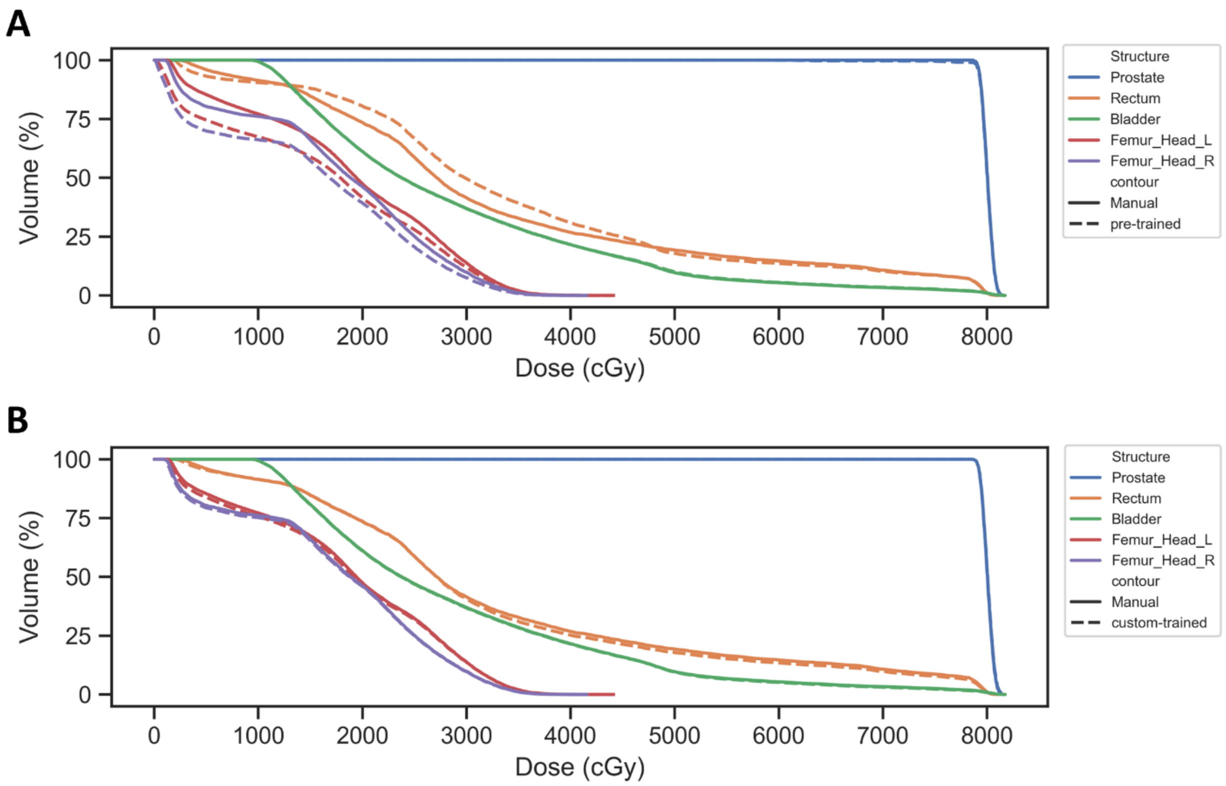


**Supplementary Figure S7**. Dose-volume histograms (DVHs) of structures from a prostate cancer clinical plan (Case 5, see Figure 3) calculated based on manual contours (solid lines) and auto-segmented contours (dotted lines) generated by (A) a vendor-pretrained model and (B) a custom-trained model. The custom-trained model showed more consistent DVHs for Prostate, Rectum, Femur_Head_L and Femur_Head_R. Abbreviations: _L, left; _R, right; Femur_Head, femoral head.

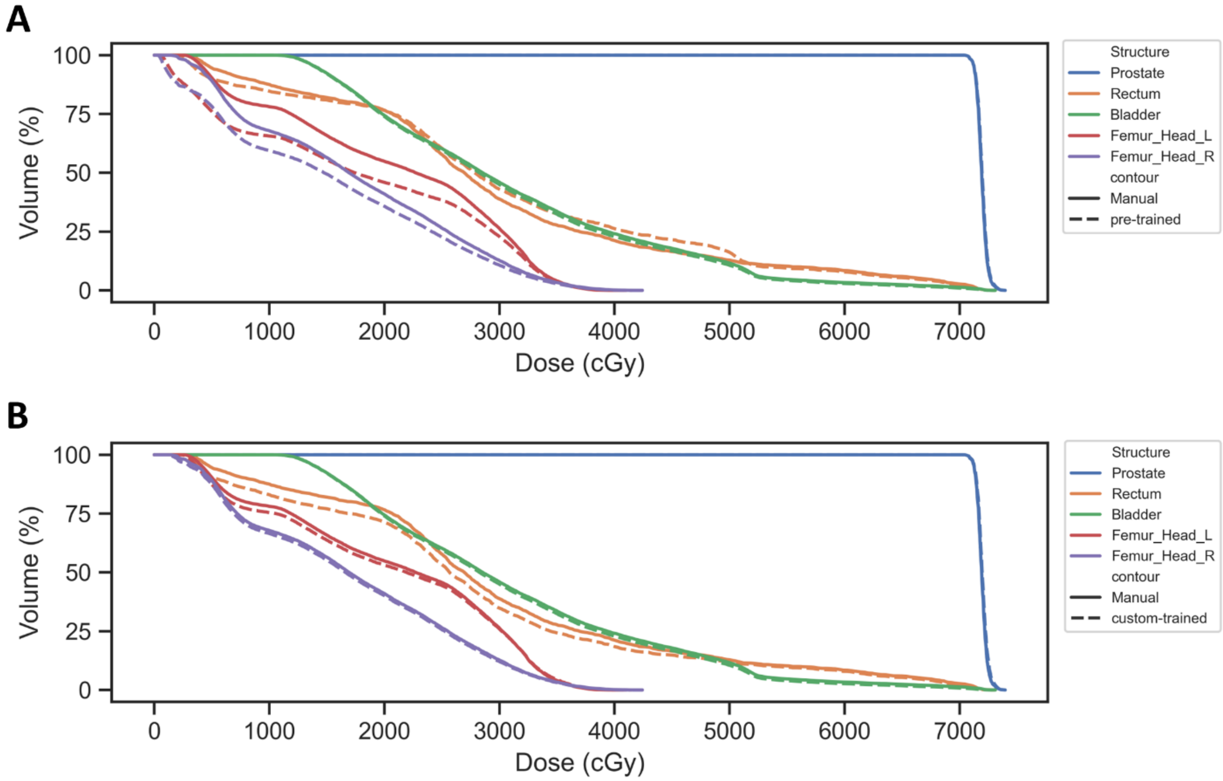


**Suppleementary Figure S8**. Dose-volume histograms (DVHs) of structures from a prostate cancer clinical plan (Case 6, see Figure 3) calculated based on manual contours (solid lines) and auto-segmented contours (dotted lines) generated by (A) a vendor-pretrained model and (B) a custom-trained model. The custom-trained model showed more consistent DVHs for Femur_Head_L and Femur_Head_R. Abbreviations: _L, left; _R, right; Femur_Head, femoral head.
